# Potency of Oral Rehydration Solution in Inducing Fluid Absorption is Related to Glucose Concentration

**DOI:** 10.1038/s41598-020-64818-3

**Published:** 2020-05-08

**Authors:** Vittoria Buccigrossi, Andrea Lo Vecchio, Eugenia Bruzzese, Carla Russo, Antonella Marano, Sara Terranova, Valentina Cioffi, Alfredo Guarino

**Affiliations:** Department of Translational Medical Science, Section of Pediatrics, University of Naples Federico II, Naples, Italy

**Keywords:** Physiology, Gastroenterology

## Abstract

Oral rehydration solutions (ORSs) is the key treatment of acute diarrhea in children, as it restores the electrolyte balance by stimulating the intestinal sodium/glucose transporter SGLT1 to induce fluid absorption. The World Health Organization (WHO) and The European Society for Paediatric Gastroenterology Hepatology and Nutrition (ESPGHAN) proposed ORSs with different chemical compositions. The main agent of childhood acute gastroenteritis is rotavirus (RV). We evaluate the effects of ORS with different concentration of glucose and sodium on RV induced secretion. Ussing chambers technique was used for electophysiology experiments to evaluate ion fluid flux. ESPGHAN ORS (sodium 60 mmol/L and glucose 111 mmol/L) induced a more potent proabsorptive effect in Caco-2 cells than WHO ORS, and this effect depended on the sodium/glucose ratio. Titration experiments showed that RV-induced fluid secretion can be reverted to a proabsorptive direction when sodium and glucose concentration fall in specific ranges, specifically 45–60 mEq/L and 80–110 mM respectively. The results were confirmed by testing commercial ORSs. These findings indicated that ORS proabsorptive potency depends on sodium and glucose concentrations. Optimal ORS composition should be tailored to reduce RV-induced ion secretion by also considering palatability. These *in vitro* data should be confirmed by clinical trials.

## Introduction

In 1978, an editorial in the Lancet reported “The discovery that sodium transport and glucose transport are coupled in the small intestine so that glucose accelerates absorption of solute and water, is potentially the most important medical advance of this century^1^”. Millions of lives of children and adults were saved by the use of a simple mixture of salt-glucose^2^.

Diarrhea is already an important cause of death primarily in children younger than 5 years. Approximately 1,3 million deaths are caused by diarrheal diseases among all ages, with the main impact on children younger than 5 years^3,4^. Fatal gastroenteritis occurs mainly in developing countries because of poor hygiene and malnutrition (300,000 deaths/year in sub-Saharan Africa), although fatality should not be neglected in the high-income countries (approximately 700 deaths/year in 2015)^5,6^. Rotavirus is the most frequent diarrheal disease causing high mortality in children younger than 5 years(150.000 deaths in 2015) with also an significant rate in older ones (200.000 deaths among all ages)^6^. The severity of gastroenteritis corresponds to the degree of dehydration defined by the amount of water and electrolyte losses. Hence, the current management of diarrhea in children involves the prevention and management of dehydration as first line therapy.

Oral rehydration solution (ORS) is a balanced mixture of glucose and electrolytes that stimulates fluid absorption and counteracts dehydration and metabolic acidosis and is used for the rehydration. ORS efficacy exploits the Na^+^/glucose cotransporter (SGLT1), located on the apical membrane of intestinal epithelial cells^7^.

The first ORS composition was jointly proposed by the United Nations Children’s Fund (UNICEF) and the World Health Organization (WHO). This formula was based on glucose/salt solution to counteract dehydration during diarrhea independently by the etiology and the age of patients^8^. This ORS is designed to counteract cholera diarrhea, because the latter was the major cause of death. WHO ORS contains Na^+^, K^+^, NaHCO_3_ in respectivity concentration of 90, 20 and 10 mEq/L plus 110 mml/L of glucose^9^. This ORS occasionally caused hypernatremia in children^10,11^; therefore, a reduced osmolarity ORS was adopted lowered both sodiumand glucose^12^. Subsequently, hyposmolar ORSs have been proposed as the standard for developed countries, which may be also indicated in nonmalnourished children in developing countries^13^.

A “salty solution”, such as WHO ORS, is less palatable than hyposmolar ORS^14^ and is not needed for non-cholera diarrhea. The European Society for Paediatric Gastroenterology, Hepatology, and Nutrition (ESPGHAN) recommends a solution containing 60 mEq/L Na^+^ for developed and developing countries^13,15^. Hyposmolar ORS is now supported by several guidelines from different parts of the world^16^. Many children with acute gastroenteritis presenting with mild to moderate dehydration and infants and younger children tend to refuse such salty solution. Hence, flavored ORS have been made available to overcome taste issues and effectively replace fluid losses.

However, the efficacy of ORS composition also depends on several factors, and our research try to find an optimal compromise between taste and fluid absorption.

The specific aim of this study was to investigate the effects of ORSs with different concentration of glucose and sodium in *in-vitro* model of ion secretion induced by Rotavirus (RV), the most frequent cause of acute gastroenteritis in children This model reflects the chloride secretion induced by the virus on intestinal epithelial cells through the measure ofelectrical parameters generated in a polar epithelium mounted in Ussing chambers. In the first half of the 20th century, biological research focused on ion channels and their activity. In 1951, Hans Ussing developed an apparatus as simple as innovative that still bears his name today^17^. The measure of ion transport across epithelial barriers was first studied in frog skin, but soon this technique was applied to the intestinal epithelium whose function is essentially based on the Na**+**, K**+** and Cl− and H_2_O equilibrium by specific channels located on the apical and basolateral membranes of the enterocytes^18^. Potential difference (PD) reflects the transepithelial passage of ions. An increase in PD is the typical effect of enterotoxins and reflects active chloride secretion. Using this model, we described the effects of RV infection on ion transport^19^. In this paper, we applied the validated *in vitro* model to describe the mechanisms of RV diarrhea in human enterocytes to test different composition of ORS. Ion movements across the epithelium are affected by ion concentrations in the intestinal lumen and generate changes in electrical parameters that are measurable.

## Results

### Effects of standard ORSs in basal condition

We first compared the effects of WHO hyposmolar ORS with ESPGHAN ORS in Caco-2 cell monolayers in basal condition.

**Figure 1:**
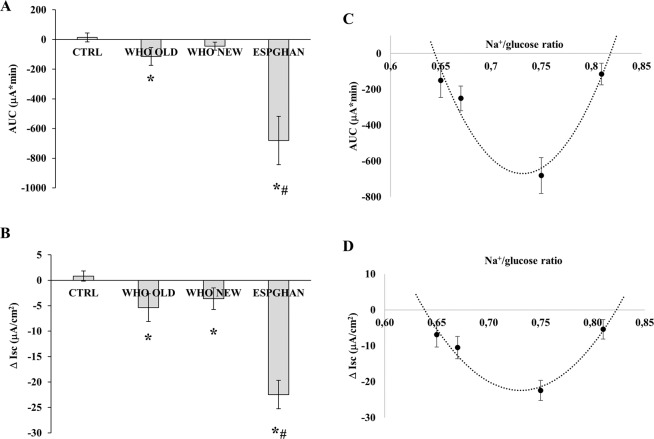
AUC (**A**) and Δ Isc (**B**) were measured in Caco-2 cell monolayers exposed to WHO original, WHO hyposmolar, and ESPGHAN ORSs. (*p < 0.05 vs CTRL; ^#^p < 0.05 vs WHO ORSs). AUC (**C**) and Δ Isc (**D**) values of four ORSs with different Na^+^/glucose ratio were used for correlation analysis. The second degree polynomial equation calculated for AUC is y = 87704x^2^ − 128305x + 46255 with R² = 0.9335 and for Δ Isc is y = 2637.8x^2^ − 3854x + 1385.4 with R² = 0.9513.

Short circuit current (Isc expressed in µA/cm^2^) and area under the curve (AUC) measured in Ussing chambers were evaluated as parameter of the secretory/absorptive response and indicating the potency of the effect, respectively. AUC and Δ Isc were negative in all conditions compared to controls indicating a pro-absorptive effect (Fig. 1A,B). The WHO hyposmolar ORS showed a lower absorptive effect than the other two ORSs in inducing fluid absorption in basal condition. A significant decrease in both AUC and Δ Isc was observed when Caco-2 cells were exposed to WHO original ORS and ESPGHAN ORS, although the latter showed a significantly higher efficiency. As shown in Fig. 1A,B, ESPGHAN ORS induced a more efficient absorption in terms of potency and the peak effect was also the highest.

Two components are essential for the pro-absorptive tone of the enterocyte, namely sodium and glucose. Both activate SGLT-1 which uses the energy from the downstream sodium ion (Na^+^) gradient created by the ATPase pump to transport glucose across the apical membrane, against an uphill glucose gradient. Hence, the Na^+^/glucose ratio is crucial for ORS effectiveness. The Na^+^/glucose ratio is 0.75 and 0.81 for ESPGHAN and original WHO ORS, respectively. We correlated the Na^+^/glucose ratio with ORS potency. In addition, we tested two commercial ORSs with the Na^+^/glucose ratio of 0.65 and 0.67. As shown in Fig. 1C,D, we found a correlation between the Na^+^/glucose ratio and AUC and Δ Isc. The second degree polynomial equation identified for AUC and Δ Isc have R^2^ of 0.9335 and 0.9513, respectively, indicating that the regression model is appropriate. According to this model, the optimal ratio for the maximum effectiveness and the range of effectiveness can be calculated. The two regression models fit together because both parabola vertices were identified at 0.73 the optimal ratio for AUC and Δ Isc parameters. The calculated ranges of effectiveness were similar, being 0.644/0.818 and 0.638/0.822 for AUC and Δ Isc, respectively.

The addition of increasing glucose concentrations induced a proabsorptive effect without difference between 80–110 mM dose (Fig. S1).

### Rotavirus-induced secretory diarrhea *in vitro*

**Figure 2:**
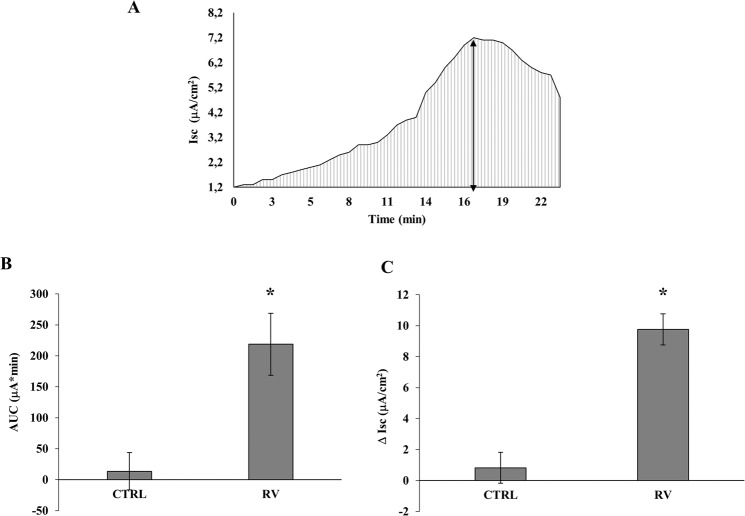
(**A**) A representative experiment showing the Isc increase monitored in Caco-2 cell monolayer infected with RV. The area under the curve (AUC, dashed area) represents the potency of the effect, the maximal increase (Δ Isc, double-headed arrow) is considered the peak effect. (**B**) AUC and (**C**) Δ Isc in RV-infected cells is significantly increased compared to controls (*p < 0.05).

Figure 2A shows a representative graph of the secretory state of Caco-2 cell monolayers induced by RV infection indicating the area under the curve (AUC) as the potency of the effect (dashed area), whereas the maximal increase (Δ Isc) is considered the peak effect (double-headed arrow). Figure 2B,C represent the significant ion secretory effect induced by RV in Caco-2 cells for both parameters.

### Effects of standard ORSs on RV-induced secretion

**Figure 3:**
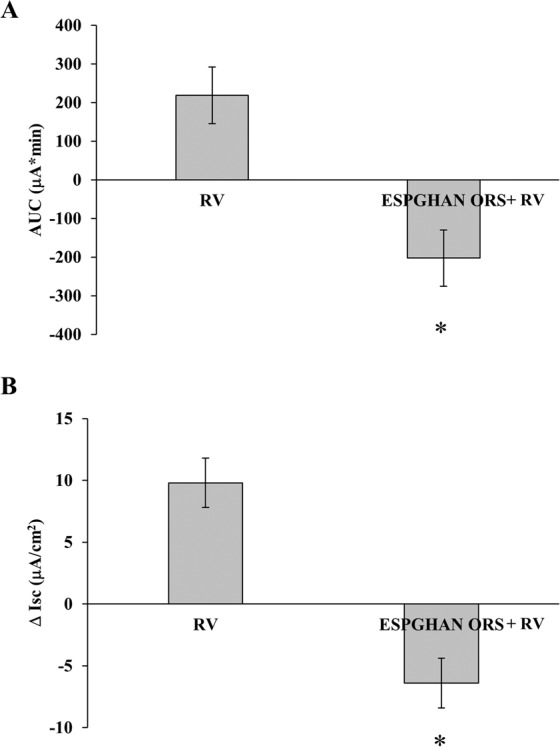
AUC (**A**) and Δ Isc (**B**) were measured in RV-infected Caco-2 cell monolayers and exposed to ESPGHAN ORSs. (*p < 0.05 vs RV).

ESPGHAN ORS significantly inhibited RV-induced ion secretion (Fig. 3A,B). The obtained negative Isc values indicated that ESPGHAN ORS is effective in reversing RV-induced ion secretion into ion absorption.

**Figure 4:**
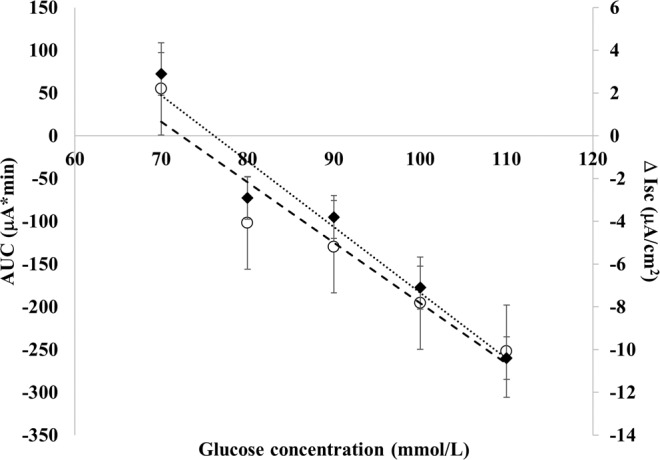
Titration analysis of increasing glucose concentration of ORSs with 60 mEq/L of sodium in RV-infected cells (ORS 1–5 in Table 1). Both AUC (○) and Δ Isc (◆) data showed a significant linear correlation (AUC y = −0,308x + 23,46 with R² = 0.9573; Δ Isc y = −7,074x + 511,8 with R² = 0.926). All results are significantly different compared to RV-infected cells (AUC = + 218.7 and Δ Isc = + 9.8; p < 0.05).

Because glucose could be a limiting factor, we performed a titration experiment using lab-prepared ORSs fixing sodium concentration at 60 mEq/L and titrating glucose concentration in a range of 70 to110 mM. In RV-infected cells, both AUC and Δ Isc data indicated a dose-response proabsorptive effect with a significant linear correlation (Fig. 4). A significant dose-response reduction of ion secretion induced by RV was observed and at higher glucose concentrations, ion secretion was converted into absorption with a dose-response effect. Based on this data, 80–110 mM of glucose concentration is associated with an optimal absorptive effect. Similar to that observed in active ion secretion, the addition of 80–110 mM of glucose in basal condition promoted ion absorption with a dose-related effect (Fig. S1).

**Figure 5:**
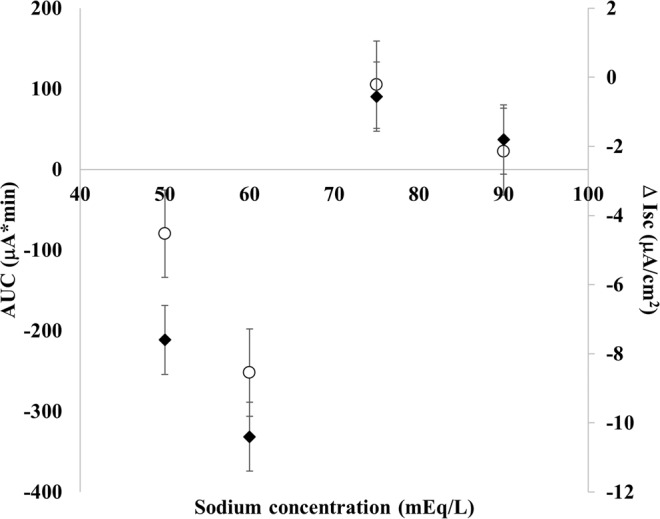
Titration analysis of increasing sodium concentration of ORSs with 110 mM of glucose in RV-infected cells (ORS 5–8 in Table 1). No significant correlation was observed for AUC (○) or Δ Isc (◆) relative to sodium doses; however, a concentration of ≤60 mEq/L induced a proabosrptive effect in RV-infected cells, whereas higher doses limited to reduce the RV-induced ion secretion. All results are significantly different to RV-infected cells (AUC = + 218.7 and Δ Isc = +9.8; p < 0.05).

Next, we investigated the optimal Na^+^ concentration range by fixing glucose at 110 mM and modifying Na^+^ concentration in the range of 50–90 mEq/L. Na^+^ concentration ≤60 mEq/L induced a proabsorptive effect in RV-infected cells, whereas higher doses were only effective in limiting the RV-induced ion secretion (Fig. 5).

### Effects of commercial ORSs in RV-induced secretion

**Figure 6:**
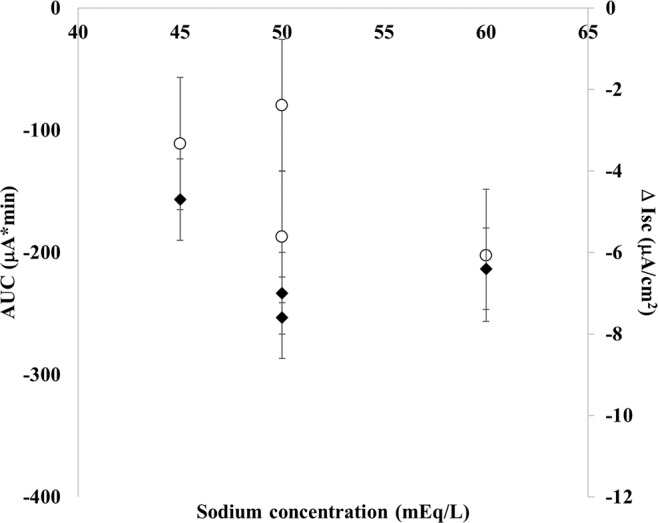
Four commercially ORSs (ORS a–d in Table 1) were tested in RV-infected cells. ORSs have glucose concentration in a range of 76–110 mM and sodium in the range of 45–60 mEq/L. All tested ORSs induce a significantly proabsorptive effect compared to RV-induced ion secretion (AUC = + 218.7 and Δ Isc = + 9.8; p < 0.05). No significant difference was observed among ORSs.

Finally, we tested commercially available ORSs in glucose ranges of 80–110 mM and Na^+^ range of 45–60 mEq/L. Data are plotted as AUC and Δ Isc in relation to Na^+^ concentration and indicate that all ORSs are effective in RV-induced ion secretion and promote proabsorptive ion flux (Fig. 6).

Taken together these data suggest a range of efficacy of the ORSs for both glucose and Na^+^. Indeed, the best results in terms of potency and peak effect are obtained for solutions with 80–110 mM of glucose and 45–60 mEq /L of Na^+^.

### Effects of standard ORSs on secretory diarrhea induced by Cholera toxin

**Figure 7:**
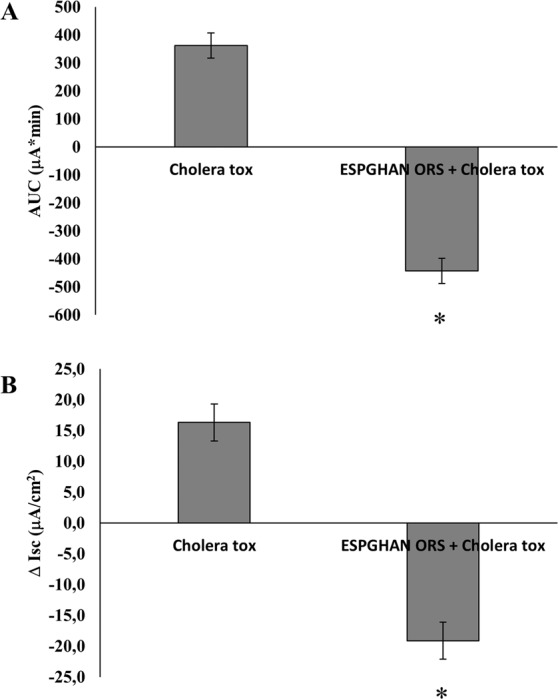
AUC (**A**) and Δ Isc (**B**) were measured in cholera toxin (6 × 10^−8^ M)-stimulated Caco-2 cell monolayers and exposed to ESPGHAN ORSs. (*p < 0.05 vs cholera toxin).

Cholera toxin induces a potent and stable increase in both AUC and Δ Isc suggesting a significant ion secretion (Fig. 7). ESPGHAN ORS was used to test transepithelial fluid transport in this condition. A complete inhibition of fluid secretion induced by cholera toxin was observed with a proaborptive effect induced by ESPGHAN ORS (Fig. 7).

## Discussion

Proximal to distal intestinal segments, from the duodenum to the distal colon, have specific mechanisms for either electrolyte absorption or secretion. Fluid transport across intestinal epithelial cells is a finely tuned process with net absorption predominating in normal conditions. However, a basal level of fluid secretion is necessary for accomplishing the digestive functions. Large water volumes are secreted and reabsorbed through the small intestinal epithelium during the digestive processes. During diarrhea, the balance is impaired, and an excess of fluids is secreted causing a net loss of water and electrolytes ultimately leading to dehydration. This is the cause of death for approximately 500 to 1 million of children annually^20^. Dehydration is strongly associated with secretory diarrhea with microorganisms including RV that produce the NSP4 enterotoxin.

The Na^+^/glucose cotransporter SGLT1 is an active pump that stoichiometrically transports two sodium ions and one glucose molecule together across the cell membrane. ORSs include both sodium and glucose because, without glucose, intestinal sodium is not actively absorbed^7^. Several antisecretory agents were evaluated to limit the secretory diarrhea in *in vitro* model without affecting ORSs efficacy^21^. The optimal concentrations of sodium and glucose in ORS are controversial. WHO and UNICEF have previously published criteria for ORS composition specifying that glucose should at least equal that of sodium within the range of 60–90 mEq/L^22^. ESPGHAN recommends an ORS with sodium concentration of 60 mEq/L and glucose concentration ranging 74–111 mmol/l^13,15,23,24^. We have used a standard validated model to test transepithelial ion transport in condition of RV chloride secretion, since RV is the most frequent and dangerous agent of childhood diarrhea worldwide.

ORSs were first designed to counteract diarrhea by *Vibrio cholerae*. More recently, other gastrointestinal agents have been recognized as major agents of gastroenteritis and benefit by ORSs effects, being diarrhea under the control of the same ion secretion mechanisms.

This is the first *in vitro* study investigating ORSs composition and their effects on transepithelial ion transport in basal and in conditions of RV infection. The basal hydro-electrolyte tone of enterocytes depends on the sodium/glucose ratio and an optimal proabsorptive effect is observed with Na^+^/glucose ratio between 0.64 and 0.82. ESPGHAN ORS has the most effective ratio (0.75).

This value corresponds to 3 sodium ions and 4 glucose molecules, which is different to the ratio of SGLT-1, i.e., 2 sodium ions and 1 glucose molecule. Because sodium is an abundant component of the extracellular medium, more glucose probably boosts pump activity.

The proabsorptive effect of ESPGHAN ORS was also observed in RV-infection model. Titration experiments allowed us to identify efficacy ranges for both sodium and glucose (80–110 mM glucose and 45–60 mEq/L of sodium). Practical guidelines of ESPGHAN for the management of gastroenteritis in children support the use of hyposmolar solution (74–111 mM glucose and 60 mEq/L sodium)^24^. Three of the four commercial ORSs chosen tested in our *in vitro* model already have a lower sodium concentration in order to improve palatability. Commercially available ORSs formulations are substantially different containing several functional factors, i.e., zinc and/or prebiotics to promote the intestinal functions. We and others have shown that zinc promotes ion absorption^25^. However, it does that in a well concentration range that has a narrow safety spectrum^26^. In addition, aromas and sweeteners are often present as ingredients in order to improve the palatability. Both palatability and osmolarity are key elements for efficacy. On the one hand, palatability is a major limit of intake, although very dehydrated children drink “salty” ORS in the attempt to replace sodium loss. On the other hand, hyposmolar solutions support net fluid absorption^27,28^. Therefore, optimal concentrations should consider both chemical composition and palatability.

Of course, our findings in an *in vitro* study on the direct interaction between ORS and the enterocyte need to be considered as a proof of concept of the critical aspect of ORS composition and may provide the basis for optimizing the composition of ORS formula. This needs to be confirmed by clinical trials. However, our data challenge the classical composition of ORS in a time in which RV is still a major agent of gastroenteritis and a killer in childhood.

## Materials and Methods

### Cell line

Caco-2 cells were used as they differentiate in mature enterocytes of upper villus forming monolayers. Cells were grown in high glucose DMEM with 10% fetal calf serum (FBS), 1% non-essential amino acids, 50 mU/ml penicillin, and 50 mg/ml streptomycin. Caco-2 cells were grown for 15–18 days after confluence on polycarbonate Snapwell filters (pore size 0,4 micron) (Costar Italia, Milan, Italy).

### Oral rehydration solutions (ORSs)

The composition of all ORSs used in this study are listed in Table 1. Caco-2 cell monolayers were stimulated with ORSs 30 minutes before RV infection.

**Table 1:** Chemical composition of ORSs used in this study.

ORS	glucose (mmol/L)	Na^+^ (mEq/L)	K^+^ (mEq/L)	Cl^−^ (mEq/L)	HCO_3_^−^ (mmol/L)	Citrate^3−^ (mmol/L)	Na/glucose ratio
WHO original ORS (1975)	111	90	20	80	30	—	0,81
WHO hyposmolar ORS (2002)	75	75	20	65	—	10	1
ESPGHAN ORS	80	60	20	50	—	10	0,75
**Lab-prepared ORSs**							
ORS 1	70	60	20	50	—	10	0,85
ORS 2	80	60	20	50	—	10	0,75
ORS 3	90	60	20	50	—	10	0,66
ORS 4	100	60	20	50	—	10	0,60
ORS 5	110	60	20	50	—	10	0,54
ORS 6	110	50	20	40	—	10	0,45
ORS 7	110	75	20	65	—	10	0,68
ORS 8	110	90	20	80	—	10	0,81
**Commercial ORSs**							
ORS a	90	60	20	37	—	10	0,67
ORS b	76	50	20	40	—	10	0,65
ORS c	110	50	20	40	—	10	0,45
ORS d	95	45	20	35	—	10	0,47

### Virus strain and infection protocol

Caco-2 cell monolayers were infected with the simian rotavirus strain SA11 (RV) at a multiplicity of infection (MOI) of 25. RV was previously activated with 20 ug/mL trypsin for 1 hour at 37 °C. Viral suspension was added to the apical side of the Caco-2 cell monolayers for 1 hour at 37 °C, and the cells were then rinsed 3 times and incubated in fetal calf serum–free medium for 1 hour. Finally, Caco-2 cells were mounted in the Ussing chamber system as described below. The model has been used to test the effects of antimicrobial drugs^29^ and has become a standard tool for studying the direct effects of drugs and toxins in human enterocytes. After infection, cell monolayers were mounted in Ussing chambers to test the effect of several ORSs. The cells were incubated with ORSs after RV infection, and electrical parameters were then measured in Ussing chambers.

### Ion transport studies

Caco-2 cells monolayers were short-circuited by a voltage clamp in Ussing chambers (Physiological Instruments, San Diego, CA). The Ussing chamber system is used to measure ion transport in polarized tissue, such as gut mucosa or in a monolayer of cells grown on permeable supports. Electrical parameters measured in Ussing systems include transmembrane voltage (Vt expressed in mV), epithelial membrane resistance expressed as tissue ionic conductance (G measured in mS/cm^2^), and short circuit current (Isc expressed in µA/cm^2^; the current required to bring Vt to 0 mV). Every 20 sec, current pulses were passed across the epithelium, and the current was recorded and transepithelial resistance (R) was calculated.

Isc was used as parameter of the secretory/absorptive response in all experimental conditions. In order to obtain a more reliable evaluation of the overall secretory/absorptive response, Isc data points between the time period of 10 to 50 mins were used to calculate the integrated response. Area under the curve (ʃIsc dt; named AUC) was calculated by a numerical integration procedure after subtraction of baseline Isc and was used as a measure of the integrated response of Caco-2 cell monolayers in experimental conditions. In addition, the peak of Isc response obtained in the time period considered (after subtraction of baseline Isc; named Δ Isc) was used as a measure of the secretory/absorptive response. Negative value of AUC and Δ Isc indicate a proabsorptive effect and positive ones indicate an active ion secretion. AUC is a parameter indicating the potency of the effect, whereas the maximal increase in Isc (Δ Isc) is considered as the peak effect.

### Statistical analysis

We used GraphPad Prism Software (San Diego, CA) to evaluate the two-tailed unpaired Student’s t test and a two-tailed paired Student’s t test to evaluate statistical significance. An alpha value of 0.05 was set for statistical significance. p values for each analysis are indicated in figure legends.

In addition, we find a correlation between biological effects observed and ORSs parameters. After plotting mean value on Excel graph, trendline tool was applied. The best fitted equation was considered for R^2^ value > 0,9. The equations and R^2^ are indicated in the text.

## Supplementary information

Supplementary information.
