# Supplementary material for: Potency of Oral Rehydration Solution in Inducing Fluid Absorption is Related to Glucose Concentration
Source: Sci Rep. 2020 May 8;10:7803. doi: 10.1038/s41598-020-64818-3 (PMC7210290; doi:10.1038/s41598-020-64818-3)
Supplement: Supplementary file 1 — Supplementary information. [file 41598_2020_64818_MOESM1_ESM.pdf]

**POTENCY OF ORAL REHYDRATION SOLUTION IN INDUCING FLUID ABSORPTION IS RELATED TO GLUCOSE CONCENTRATION**

Vittoria Buccigrossi[1], Andrea Lo Vecchio[1], Eugenia Bruzzese[1], Carla Russo[1], Antonella Marano[1], Sara Terranova[1], Valentina Cioffi[1] and Alfredo Guarino[1*]

**SUPPLEMENTARY FIGURE**

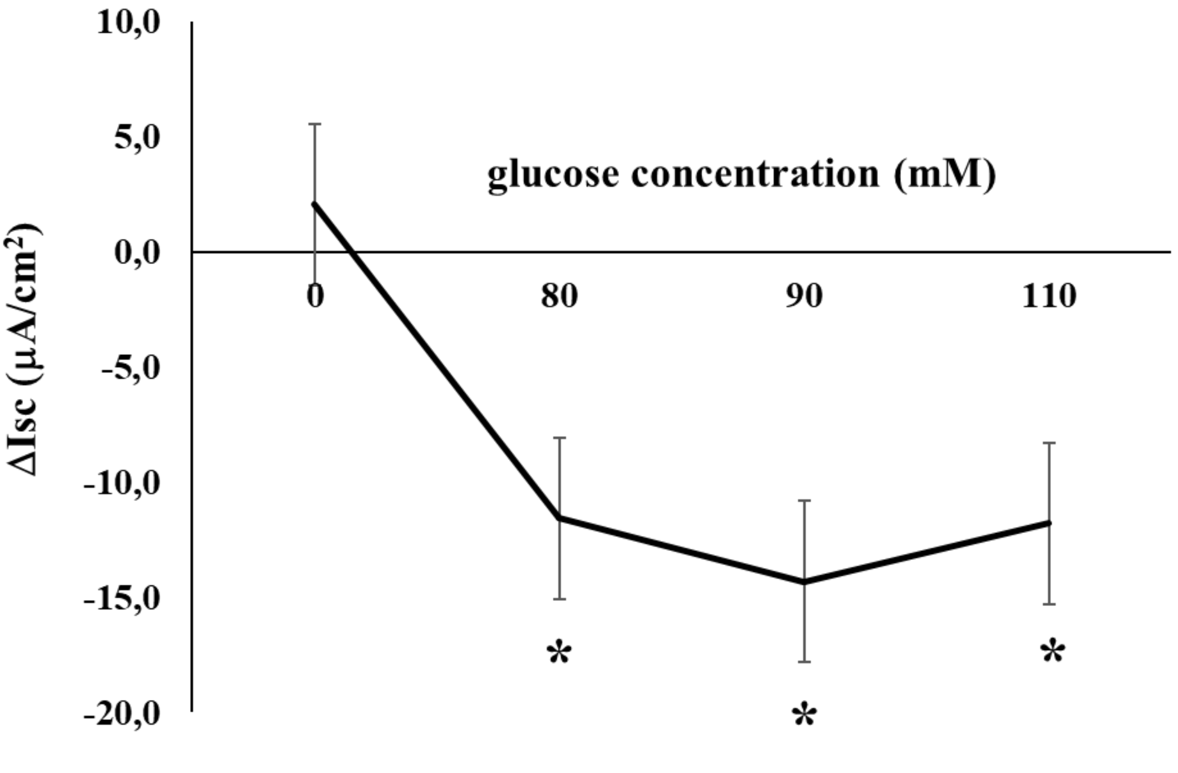


**Fig. 1S.** Titration analysis of increasing glucose concentration. Δ Isc data showed a proabsorptive effects without dose-related dependence. * $p<0.05$ vs 0 mM.
